# When Cells Suffocate: Autophagy in Cancer and Immune Cells under Low Oxygen

**DOI:** 10.1155/2011/470597

**Published:** 2011-11-29

**Authors:** Katrin Schlie, Jaeline E. Spowart, Luke R. K. Hughson, Katelin N. Townsend, Julian J. Lum

**Affiliations:** ^1^Deeley Research Centre, BC Cancer Agency, 2410 Lee Avenue, Victoria, BC, Canada V8R 6V5; ^2^Department of Biochemistry and Microbiology, University of Victoria, Petch Bldg Ring Road, Victoria, BC, Canada V8P 5C2

## Abstract

Hypoxia is a signature feature of growing tumors. This cellular state creates an inhospitable condition that impedes the growth and function of all cells within the immediate and surrounding tumor microenvironment. To adapt to hypoxia, cells activate autophagy and undergo a metabolic shift increasing the cellular dependency on anaerobic metabolism. Autophagy upregulation in cancer cells liberates nutrients, decreases the buildup of reactive oxygen species, and aids in the clearance of misfolded proteins. Together, these features impart a survival advantage for cancer cells in the tumor microenvironment. This observation has led to intense research efforts focused on developing autophagy-modulating drugs for cancer patient treatment. However, other cells that infiltrate the tumor environment such as immune cells also encounter hypoxia likely resulting in hypoxia-induced autophagy. In light of the fact that autophagy is crucial for immune cell proliferation as well as their effector functions such as antigen presentation and T cell-mediated killing of tumor cells, anticancer treatment strategies based on autophagy modulation will need to consider the impact of autophagy on the immune system.

## 1. Introduction

In many tumors, cell growth and proliferation exceeds the development of local vasculature supplying oxygen and nutrients. As a result, tumors form disorganized angiogenic vessels that cause the percent of oxygen within the tumor to range heterogeneously from anoxic (<0.5% O_2_) and hypoxic (0.5–1.5% O_2_) to normoxic (>1.5% O_2_) levels [1, 2]. Cancer cells in close proximity to vasculature contribute to tumor hypoxia by rapidly utilizing oxygen and nutrients that arrive at the tumor site. This can result in either chronic or cycling hypoxia depending on how quickly cancer cells consume oxygen once new vascular networks are formed [3, 4]. To circumvent the effects of oxygen deprivation, the transcription factor hypoxia inducible factor-1*α* (HIF-1*α*) is stabilized in cells under hypoxia. HIF-1*α* allows for adaptation to hypoxia by promoting a metabolic switch from oxidative phosphorylation to glycolysis and by initiating angiogenesis [5]. Collectively, the hypoxic and nutrient-depleted tumor microenvironment impacts the metabolism, survival, and function of all cells exposed to it.

As a result of hypoxic stress, cells in the tumor microenvironment activate autophagy, a cell survival process that degrades and recycles cellular constituents. Autophagy can be induced by various stressors including nutrient starvation, growth factor withdrawal, hypoxia, and chemotherapeutic stress [6–9]. During autophagy, selective or bulk portions of cytoplasm, including whole organelles, are sequestered in double-membraned vacuoles called autophagosomes. These structures fuse with lysosomes to form autophagolysosomes, the site of degradation for the sequestered cargo. Metabolites within the autophagolysosome are liberated by vacuolar permeases, which efflux amino acids and other nutrients from the degradative compartment back into the cytosol where they are recycled as biosynthetic precursors, or as substrates that are oxidized to support bioenergetics [10]. The biochemical steps of autophagy initiation, autophagosome elongation, and recycling have been elegantly documented by numerous investigators and are the subject of several recent reviews [11–13]. Here, we focus on the activation and consequences of autophagy under the condition of low oxygen.

In the context of cancer, activation of autophagy by hypoxia impacts tumorigenesis, cancer cell viability, and likely antitumor immunity. Despite the evidence that autophagy functions as a tumor suppressor in hypoxic tissues [14], the role of autophagy in maintaining the integrity of established tumors is controversial. One model suggests that cancer cells rely on autophagy as an adaptive survival mechanism to offset stressors in the tumor microenvironment [15, 16]. An alternative model proposes that prolonged autophagy leads to death by apoptosis [17, 18] or initiates a form of nonapoptotic-programmed cell death, called “type II” or “autophagic” cell death [19]. The latter form of cell death is poorly defined mechanistically, and its existence and definition have been contested in the literature as it is unclear whether cells that die in this manner are doing so by autophagy or simply with features of autophagy [20]. The role of autophagy in cancer pathology reaches an additional level of complexity when tumor-infiltrating immune cells are considered. Immune cells traffic to tumors where they are known to recognize and kill neoplastic tissues [21–23]. Given that immune cells in the tumor microenvironment are also exposed to low oxygen, it is likely that they too become autophagic. Whether the activation of autophagy helps or hinders immune cell function is currently unknown. Therefore, understanding the physiological consequences of autophagy in different cell types in the tumor microenvironment is critical when considering therapies that target autophagy.

## 2. Hypoxia-Induced Autophagy in Tumor Cells

### 2.1. Conflicting Roles of Hypoxia-Induced Autophagy in Tumor Cells

**Figure 1:**
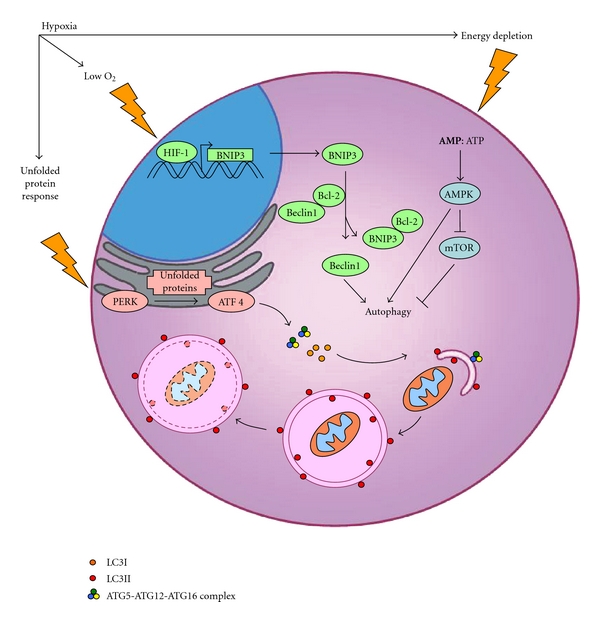
Pathways of autophagy induction by hypoxia. During hypoxia, autophagy is activated by sensors that detect low oxygen, unfolded proteins, and energy depletion. *Low *O_2_ *: * in the absence of oxygen, the alpha subunit of the transcription factor HIF-1 is stabilized resulting in the expression of the regulatory proteins BNIP3 and BNIP3L. BNIP3 and BNIP3L interact with Bcl-2 and Bcl-xL proteins that inhibit Beclin1, a key regulator of autophagy induction. The resulting liberation of Beclin1 leads to the activation of autophagy [8]. (BNIP3L and Bcl-xL are not shown in the figure). *Unfolded Protein Response:* autophagy may be induced during hypoxia as a result of signals generated by the unfolded protein response in the endoplasmic reticulum. PERK detects unfolded proteins and induces ATF4 to upregulate the expression of the essential autophagy genes LC3 and ATG5 [28, 29, 33]. LC3 I is processed to its active form, LC3 II, and trafficked with the ATG5-ATG12-ATG16 complex to the elongating autophagosome. *Energy Depletion:* increases in the intracellular ratio of AMP to ATP during hypoxia activates AMPK, an energy sensing switch that activates autophagy both directly and indirectly by inhibiting mTOR [14, 101].

The role of autophagy in hypoxic tumors is controversial given opposing studies that show a correlation of autophagy with enhanced [8, 24, 25] or decreased tumor cell survival [14, 26]. Some of these discrepancies may in part be due to cell type and/or the activating autophagy signal. Along these lines, hypoxia-induced autophagy has been demonstrated to promote tumor survival by several mechanisms, including the removal of damaged mitochondria that produce cytotoxic reactive oxygen species (ROS) [24, 27] and the degradation of harmful protein aggregates [28, 29]. In addition, autophagy is believed to sustain the energetic needs of the cell during hypoxia and nutrient withdrawal by liberating metabolites that can be oxidized to generate ATP. One way that cells sense and adapt to their energetic requirements is through the energy sensor adenosine monophosphate-activated protein kinase (AMPK). As the intracellular ratio of AMP to ATP increases, AMPK activity promotes autophagy induction serving as a means to prevent prolonged energy crisis and eventual cell death (Figure 1). By contrast, hypoxia-induced autophagy has also been demonstrated to suppress tumor cell growth and survival [14]. This suppression likely results from a combination of prolonged self-eating, the activation of cell death associated with features of autophagy, and the induction of apoptosis by autophagy-related proteins [18, 30]. Knowing when and how autophagy orchestrates cancer cell survival when considering the complex hypoxic milieu in the tumor microenvironment is therefore essential if appropriately tailored therapies are to be employed with the goal of crippling tumor cell survival. 

#### 2.1.1. Autophagy as a Survival Pathway for Tumor Cells under Hypoxia

Although autophagy can have negative implications on tumor growth, it appears that the primary function of autophagy is to help cancer cells cope with environmental stress. During hypoxia, autophagy has been demonstrated to be essential for the survival of various cell types including human colon and prostate carcinoma cell lines [8]. Elucidation of how autophagy was induced in these cells revealed that autophagy activation was dependent on the HIF-1-BNIP3/BNIP3L-Beclin1 interacting axis (Figure 1). HIF-1*α* target genes include the autophagy regulatory proteins BNIP3 (Bcl-2/adenovirus E1B 19-kDa interacting protein 3) and BNIP3L (BNIP3-like protein). Upregulation of BNIP3 and BNIP3L during hypoxia has been shown to induce the selective degradation of mitochondria by autophagy (so-called mitophagy), a process that promotes cell survival by reducing the generation of DNA damaging ROS by dysfunctional mitochondria [27]. Mitophagy appears to be important for promoting survival during hypoxia in hepatocellular tumor spheroids which have a natural hypoxic gradient [24]. Here, HIF-1*α*-induced BNIP3 expression presumably led to enhanced mitophagy, and when autophagy was inhibited chemically, increased tumor cell death occurred. Another report has shown that a BNIP3/BNIP3L-independent form of HIF-1*α*-induced autophagy can initiate bulk degradation, but not mitophagy, under hypoxia in concert with platelet-derived growth factor receptor family signalling. This latter form of hypoxia-induced autophagy was also important for tumor cell survival under hypoxia [31]. 

Previously, it was shown that p53-deficient tumor cells adapt to and survive in hypoxic environments superior to p53-competent tumor cells [32]. A more recent study shows that the resistance of these tumor cells to hypoxia may be in part due to autophagy that is activated by the absence of p53. Tasdemir et al. were the first to report that the absence of p53 resulted in induction of autophagy via AMPK in a variety of cell lines. Furthermore, this induction is specifically dependent on the absence of cytoplasmic p53 which is canonically responsible for inhibiting autophagy [25]. When p53-deficient cells were subjected to metabolic stress (nutrient deprivation and hypoxia), these cells had a survival advantage over their wild-type counter parts, but this advantage was abolished if autophagy was inhibited. Interestingly, addition of the mitochondrial substrate methyl-pyruvate only partially rescued the wild-type cells subjected to metabolic stress, indicating that autophagy contributes to prosurvival functions beyond immediate nutrient mobilization. 

During hypoxia, the unfolded protein response (UPR) is initiated because oxygen is required for the formation of disulfide bonds and the maturation of proteins destined to be secreted or incorporated into the plasma membrane [33] (Figure 1). Autophagy can also promote tumor survival during hypoxia by degrading misfolded protein aggregates that accumulate in the endoplasmic reticulum. In breast cancer cells, UPR-dependent upregulation of the activating transcription factor 4 (ATF4) has been shown to induce cytoprotective autophagy in hypoxic cells [28]. Here, inhibition of autophagy led to increased apoptosis and decreased viability, indicating that autophagy plays a role in preventing cell death. UPR signalling has been shown to be required for sustained autophagy under hypoxia in colorectal carcinoma and glioma cells which were not able to maintain the LC3 levels required for autophagy under prolonged hypoxia when the ATF4-activating protein, PKR-Like ER Kinase (PERK) (Figure 1), was disrupted [29].

Despite a diverse set of signals which can activate autophagy, the acute responses to cellular stress primarily serve to maintain bioenergetic integrity and survival. This is supported by reports which have demonstrated that autophagy frees up nutrients that are utilized to generate ATP and support cell survival [6, 7, 25, 34–36]. However, little is known about the source and destination of metabolites that are liberated by autophagic degradation during hypoxia. It is possible that metabolites are either oxidized in the mitochondria, or instead held on reserve to be utilized by oxidative phosphorylation once normoxic conditions are restored. These are not mutually exclusive possibilities and could explain how tumor cells evade treatment-induced apoptosis and subsequently might contribute to tumor recurrence [29, 37–39].

Clinical evidence also suggests that tumors utilize autophagy to survive and proliferate during hypoxia. When assessed alone, high Beclin 1 expression in advanced human nasopharyngeal carcinoma samples correlated with poor patient survival [40]. This trend was also found when assessing Beclin 1 expression in combination with HIF-1*α*, indicating that hypoxia-induced activation of Beclin 1 and autophagy may drive carcinoma cells to survive treatment and potentially lead to reoccurrence. Similar results have been reported from a study investigating tissue samples from colorectal adenocarcinoma [41]. In this study, patients with either extensive increased or decreased expression of Beclin 1 had poorer survival than patients with normal levels of Beclin 1 expression. The authors speculated that tumors with low expression of Beclin 1 had an advantage because decreased Beclin 1 reduced interactions with anti-apoptotic proteins, whereas tumors with high expression of Beclin 1 activated autophagy, in turn allowing tumor cells to overcome conditions that would otherwise result in cell death. It was also found that extensive overexpression of Beclin 1 was correlated with markers of both the presence of hypoxia and cellular adaptation to hypoxia, providing further evidence supporting the favourable role of hypoxia-induced autophagy in tumor cell survival. 

#### 2.1.2. Autophagy as a Negative Regulator of Tumor Survival and Growth

As some cells have been observed to die with structural features of autophagy in the absence of apoptotic markers, some researchers have proposed that cells can die by autophagy, a term known as autophagic cell death [19]. However, considering that autophagy is primarily a cell survival process, it is likely that in the majority of such cases, the cells are actually using autophagy as a last attempt to survive, as opposed to actually dying by autophagy [20]. Despite this, the idea that cancer cells can undergo death by excessive autophagy has attracted attention from various groups looking to exploit autophagy as a way to kill tumors [14, 26]. Hypoxia-induced autophagy has been demonstrated to reduce survival in several cell types including glioma, breast cancer, and human embryonic kidney cells independent of apoptosis [26]. In all of these cell types, autophagy-dependent cell death was observed following prolonged exposure to hypoxia. This decrease in tumor cell survival was abrogated when autophagy was inhibited, suggesting that cell death associated with autophagy may have been responsible for the reduction in cellular viability. However, further investigation into the mechanism of autophagy induction in this study revealed that it involved BNIP3. Given that BNIP3-induced autophagy is associated with the activation of mitophagy, a process that is generally cytoprotective during hypoxia [27], it appears that the duration of hypoxia treatment may have caused cells to eat themselves to death or deplete mitochondria to levels insufficient for survival. Despite the fact that the cancer cells mentioned above initially survived by autophagy [26], this process is self-limiting and could be viewed as a failed attempt to survive rather than a classical cell death pathway when cells succumb to death. 

An alternative explanation for why autophagy negatively impacts cell survival during hypoxia is that specific signalling events may occur in certain cell types, triggering cell death associated with autophagy. Experimental evidence in support of this model demonstrated that mouse embryo fibroblasts (MEFs) cultured under hypoxia and nutrient replete conditions activate autophagy through AMPK signalling, independent of HIF-1*α*, resulting in cell death [14]. Interestingly, if ATG5 was knocked out, the cells had increased viability under hypoxia. In this study, autophagy competent MEFs were less glycolytic than ATG5 knockout MEFs, indicating that the switch to glycolysis is important for hypoxic cells to survive. One possibility is that metabolites liberated by autophagy in these cells are fed into oxidative phosphorylation, hindering the switch to glycolysis and thus negatively affecting the viability of these cells under hypoxia. This hypothesis is supported by the fact that mitochondria are not specifically degraded without HIF-1*α* activation [27]. Therefore, aberrant oxidation of autophagy-derived nutrients in the mitochondria may contribute to ROS-mediated cell death in these cells. 

In addition to slowing tumor growth by triggering excessive self-eating and death associated with autophagy, hypoxia-induced autophagy can also activate apoptotic cell death. The positive effect of ATG5 deletion in the previous example [14] might also be explained by a role of ATG5 to drive apoptosis. One study demonstrated that ATG5 cleavage and translocation into the mitochondria triggers cytochrome C release and caspase cleavage [18, 42]. The cross-talk between autophagy and apoptosis during hypoxia is further highlighted in models of transformed rat epithelia, where overexpression of the essential autophagy gene, Beclin 1, results in caspase dependent cell death [17]. It is possible that Beclin 1 exerts its prodeath function through its caspase-mediated cleavage [30, 43] or by binding to Bcl-2 or Bcl-xL, sequestering these proteins and preventing them from exerting their prosurvival functions [44] (Figure 1). 

#### 2.1.3. Autophagy as a Method of Treatment Resistance under Hypoxia

Hypoxic tumor cells present a particular challenge when treating cancer patients because these cells are often more resistant to many forms of anticancer therapy than normoxic cells [45]. The involvement of autophagy in mediating this resistance was shown by inhibiting or knocking down autophagy in tumor cells treated with the chemotherapy drug cisplatin under hypoxia. Abrogation of autophagy reduced tumor cell viability to levels similar to those observed when cells were treated with cisplatin under normoxia [46]. Interestingly, the synergistic effect between chemotherapy treatment and autophagy inhibition is not always observed under normoxia, but rather only under hypoxia. An example of a drug for which this has been shown is *N*-(4-hydroxyphenyl)retinamide [47]. This phenomenon brings up an important consideration when testing for cancer treatment sensitivity by autophagy inhibition. In light of the fact that solid tumors will experience some degree of hypoxia and these cells are often the most resistant to treatment, autophagy inhibition and cancer treatment combination experiments should be performed *in vitro* not only under normoxic conditions, but also under low oxygen. In addition, scenarios of cycling and chronic hypoxia which are present *in vivo* may also need to be taken into consideration.

Not all anticancer agents display reduced cytotoxicity towards hypoxic cells. In fact, some drugs, such as Bortezomib, have been demonstrated to have increased efficacy under hypoxia [48]. Bortezomib is a proteasome inhibitor that induces autophagy by stimulating the unfolded protein response. Inhibition of autophagy concurrently with Bortezomib treatment under hypoxia resulted in greater apoptosis of cervical carcinoma cells [48]. Coupling a proteasome inhibitor with an autophagy inhibitor when treating hypoxic tumors is very attractive given that hypoxia results in misfolded proteins and inhibiting both the proteasome and autophagy blocks the cell's two key methods for disposing of such proteins. 

Another difficulty with the treatment of hypoxic tumors is their increased resistance to radiation therapy. Interestingly, this resistance can be circumvented by inhibiting autophagy as has been shown in mouse xenograft models of colorectal carcinoma and glioma [29]. This is an exciting result since hypoxic cells are notorious for being resistant to radiation. A simple method for sensitizing such cells would be of significant clinical benefit.

Considering the results discussed above, cancer cells predominantly use autophagy as a survival mechanism under hypoxia. Therefore, inhibiting autophagy in hypoxic cancer cells may be exploited to increase treatment efficacy. However, accumulating evidence suggests that autophagy also plays an important role in mediating immune cell survival, development, and function [49, 50]. Given that a robust antitumor immune response is associated with increased patient survival in several cancer types, further consideration must be given to the immunological consequence of autophagy inhibition in the tumor microenvironment. 

## 3. Autophagy in Immune Cells in a Hypoxic Tumor Environment

### 3.1. Immunosurveillance of Tumor Growth by Innate and Adaptive Immune Cells

The tumor microenvironment is inhospitable not only for tumor cells but also immune cells that traffic to the tumor bed. This hostility is compounded by immunosuppressive molecules secreted from the tumor cells into the environment, including nitric oxide, adenosine, and suppressive cytokines [51–53]. In spite of these hurdles, multiple cell types within the innate and adaptive immune system are capable of recognizing and eliminating tumor cells.

Professional antigen presenting cells (APCs) such as macrophages, B cells, and dendritic cells (DCs) display peptides phagocytosed in the tumor environment on major histocompatibility complex (MHC) molecules for recognition by T cells. Phagocytosed tumor antigens are either presented on MHC class II molecules which activate helper CD4+ T cells, or the antigen is cross-presented onto MHC class I molecules to activate antitumor cytotoxic CD8+ T cells [54]. Overall, the innate immune system is linked to the adaptive immune system through APC presentation of tumor-specific or associated antigens in combination with costimulatory signals, allowing for antitumor activity.

Because the immune system must directly interact with tumors to facilitate cell death, its components experience hypoxia in the tumor microenvironment. Immune cells need to adjust their metabolic dependency once they have reached the tumor and may also use autophagy to enhance their survival. In the following section we review the ways autophagy might influence immune responses under low oxygen conditions.

### 3.2. The Role of Autophagy in Antitumor Innate Immune Cells Infiltrating into Hypoxic Tumor Sites

Tumors are often associated with chronic inflammation, a host response to deal with damaged tissues or infection [55]. Neutrophils are the first innate cell type to migrate to the inflammatory site where they promote inflammation and activate macrophages and DCs [56]. Unlike other immune cell types, neutrophils are programmed with a high glycolytic rate making them especially resistant to hypoxia, a property relevant for their performance in hypoxic tumors [57]. Autophagy has been shown to mediate programmed necrotic cell death of neutrophils under inflammation, and this late stage induction of cell death might serve as a mechanism to limit inflammation [58]. In the tumor environment this mechanism might impact one of the earliest antitumor immune responses by impairing neutrophil survival.

**Figure 2:**
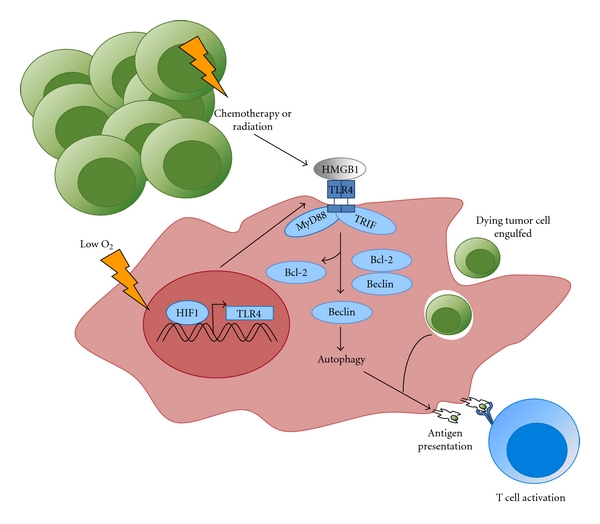
Proposed roles for hypoxia-induced autophagy in APCs. During low oxygen HIF-1*α* initiates TLR4 transcription [102]. TLR4 can then be engaged by HMGB1 (high-mobility group box-1 protein), a danger-associated molecular pattern molecule released from dying tumor cells after chemotherapy or radiation resulting in activation of the APC [103]. The engagement of TLR4 results in signalling through adaptor molecules MyD88 (myeloid differentiation primary response gene 88) and TRIF (TIR-domain-containing adapter-inducing interferon-*β*) which both inhibit the interaction of Bcl-2 with Beclin 1 to induce autophagy [64, 65]. Autophagy can then aid in processes such as improved phagosomal degradation and MHC presentation of tumor antigens for activation of the immune response [66, 67].

In contrast to neutrophils, APCs must metabolically adapt to low oxygen through stabilization of HIF-1*α* to induce increased expression of glucose transporters and glycolytic enzymes as well as limiting oxygen consuming oxidative phosphorylation [5]. As a consequence of hypoxia, APCs, such as macrophages and DCs, have decreased phagocytosis, reduced migratory capacity, and increased production of proangiogenic and proinflammatory cytokines such as vascular endothelial growth factor, tumor necrosis factor alpha, interleukin-1 (IL-1), and IL-12 [59–61]. For example, a recent study showed that DCs cultured under low oxygen resulted in skewing from a type 1 helper CD4+ T cell phenotype to a type 2 helper CD4+ T cell phenotype, which is more immunosuppressive and less beneficial for tumor killing [62]. While hypoxia is involved in dampening APC activity, autophagy may contribute to survival of APCs under these conditions. Importantly, Naldini et al. found that culturing DCs under low oxygen resulted in the stabilization of HIF-1*α* which initiated BNIP3 expression and promoted survival of mature DCs, possibly due to induction of autophagy [63]. In APCs infiltrating a tumor, hypoxia-induced autophagy can also occur via different signalling mechanisms such as toll-like receptor (TLR) signalling [64, 65]. A proposed mechanism of how hypoxia-induced autophagy influences APC function is shown in Figure 2.

In summary, autophagy plays a cell death role in neutrophils which may serve as an anti-inflammatory mechanism to regulate neutrophils in a hypoxic tumor. In contrast, autophagy in tumor-infiltrating APCs is involved in survival, likely by liberation of nutrients required to support the energy demands of an activated cell and is important for the cell's antigen presentation capabilities [66, 67]. The induction of autophagy under hypoxia may serve to improve metabolism under these conditions and allow for maintenance of proper antitumor immune function. Considering the fact that autophagy was shown to be important for the process of antigen presentation, it may be involved in positive effects of APC presence within tumors such as activation of T cells through improved MHC expression.

### 3.3. Autophagy as a Modulator for T Cell-Mediated Immunity under Hypoxic Conditions

Many immune therapies modulate T cell effector function as a means to increase antitumor immunity [22, 68–71]. T cells are able to identify tumor cells via tumor associated antigens, which are derived from proteins either mutated or not normally exposed to the immune system. Antigen recognition and stimulation of T cells in the tumor environment occurs in the presence of immunosuppressive factors that impact the efficacy of the T cell-mediated antitumor response. In the next section, we describe how hypoxia affects the fate of T cells in two ways. First, the immunosuppressive role of hypoxia will be considered. Second, hypoxia will be examined as a potent activator of autophagy and modulator of metabolism, which helps intratumoral T cells exposed to the hypoxic tumor milieu to survive.

#### 3.3.1. Signalling Events and Metabolism in Activated T Cells

**Figure 3:**
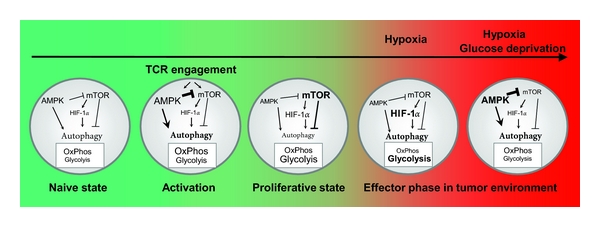
Signalling events and metabolic consequences in T cells. In the absence of activation signals, naïve cells utilize oxidative phosphorylation (OxPhos) as the main source of cellular bioenergetics. This is in part supported by basal levels of autophagy necessary to maintain energy and protein turnover homeostasis. Within the first hour of T cell activation, AMPK activity predominates leading to suppression of mTOR which results in upregulation of glycolysis, OxPhos as well as autophagy. Together these pathways provide the initial wave of bioenergetics to support growth. During the transition to the proliferative state, AMPK signalling declines and mTOR activity increases which in turn enforces nutrient uptake and glycolysis. As effector T cells enter the hypoxic tumor microenvironment, HIF-1*α* accumulation reduces OxPhos and further augments glucose-dependent metabolism. At later stages of prolonged hypoxia, exhaustion of glucose activates AMPK leading to mTOR suppression and the activation of autophagy-dependent survival and effector T cell antitumor function.

Newly activated T cells metabolically adapt to facilitate growth and proliferation. This process is modulated by several signalling pathways, including AMPK, HIF-1*α*, and mTOR (mammalian target of rapamycin) (Figure 3) [72–74]. Within one minute after engagement of the T cell receptor, AMPK becomes fully activated due to calcium influx into the cytoplasm [72]. AMPK upregulates ATP producing processes including fatty acid oxidation and glycolysis supported by elevated glucose influx, while also inhibiting ATP-consuming processes including protein, glycogen, and fatty acid synthesis. Moreover, AMPK activates autophagy by suppressing mTOR, the master regulator of protein synthesis, growth and proliferation, and suppressor of autophagy [74, 75]. This has the dual benefit of supplying cells with biosynthetic precursors via glycolysis and maximal energy production derived from oxidation of carbon substrates in the mitochondria. One assumption is that early activation of AMPK is crucial for increasing the cellular pool of ATP as a means to prepare T cells for the upcoming bioenergetic and biosynthetic demands of rapid proliferation. Following the first hour after T cell receptor engagement, AMPK activity subsides, resulting in the abrogation of the AMPK-mediated suppression of mTOR [76]. To sustain high levels of growth, T cells secrete proliferative cytokines like IL-2 that act in a paracrine manner to activate mTOR signalling. This second wave of signal transduction reinforces the cells to sustain a high metabolic state of upregulated glycolysis [77]. The dependency of activated T cells on glycolysis allows for energy production even in regions of low oxygen levels such as in inflamed tissues or hypoxic tumors. Additionally, glycolytic intermediates are the predominant sources of metabolites used in nucleic acid and lipid biosynthesis during periods of rapid T cell growth and proliferation [78, 79]. 

#### 3.3.2. Impact of Hypoxia on T Lymphocytes

Upon entry of T cells into sites of hypoxia, HIF-1*α* accumulation enables the cells to survive within regions of low oxygen, but also suppresses T cell function to avoid an excessive immune response that potentially damages healthy tissue. Unfortunately, this suppression of T cell function may also occur in a hypoxic tumor environment, leading to a down regulated antitumor immune response. Evidence for the suppressive activity of hypoxia on T cells was first described in mouse lymphoid cells. This initial study showed that incubation of naïve T cells under hypoxia prior to their activation led to the reduction of the proliferative cytokine IL-2 [80]. Subsequent investigations of mouse T cells in which HIF-1*α* was genetically knocked down revealed that this cytokine suppression was dependent on HIF-1*α* expression [81]. Importantly, activation of T cells under low oxygen conditions was also shown to impair T cell proliferation [81]. These functional T cell defects can partly be explained by interference of HIF-1*α* with calcium signalling, one of the early events initiated by T cell receptor engagement [82, 83].

In addition to the direct suppression of T cell activity, HIF-1*α* was also demonstrated to indirectly modulate T cell mediated killing by influencing the differentiation of CD4+ T cells [84, 85]. The four functional CD4+ subsets Th1, Th2, Th17, and T regulatory cells have different levels of glycolytic activity. HIF-1*α* controls differentiation into these effector subsets by modulating their metabolic signatures [85]. Moreover, lymphoid cells were shown to upregulate FoxP3 when cultured under hypoxia as a result of HIF-1*α* stabilization [84]. FoxP3 is the transcription factor driving CD4+ T cell differentiation towards T regulatory cells, which is known to attenuate T cell-mediated immune responses [86]. Interestingly, this transcription factor has also been identified as an important immunosuppressive factor especially in antitumor immunity [87]. The observation that T regulatory cells are even more efficient in their suppressive activity under low oxygen conditions once more underscores the immunosuppressive nature of hypoxia [84].

#### 3.3.3. Survival Mechanisms of T Cells under Hypoxia

In addition to the various roles of HIF-1*α* on the suppression of T cell-mediated immune responses, it has also been shown to act as a cell survival factor in T cells. For example, stabilization of HIF-1*α* represses activation-induced cell death (AICD), a process that is mediated by the Fas-Fas ligand death receptor pathway [88]. This finding is especially important for antitumor T cells, because one mechanism of the tumor's immune evasion is an elevated expression of Fas ligand for the induction of tumor-mediated AICD [89].

As mentioned in the sections above, HIF-1*α* expression induces tumor cell autophagy (Figure 1). However, the role of HIF-1*α*-dependent activation of autophagy in lymphoid cells has not yet been reported. In T cells, autophagy is activated upon T cell receptor engagement in both CD4+ and CD8+ subtypes [90–92]. The knockdown of the essential autophagy-related genes ATG5 or ATG7 during T cell receptor stimulation leads to a significant decrease in cellular proliferation demonstrating the importance of autophagy during T cell activation [91, 92]. At later time points after activation, autophagy-deficient T cells undergo apoptosis at an unusually high degree, which may be due to a defect in the initial signalling events of T cell activation. Support for this idea comes from the finding that T cell stimulation by phorbol myristate acetate (PMA) and Ionomycin, which bypasses T cell receptor signalling, results in almost normal proliferation rates of autophagy-incompetent T cells [91]. Interestingly, PMA/Ionomycin treatment triggers calcium influx into the cytoplasm which, as previously mentioned, activates AMPK during the initial phase of T cell activation. However, in which way autophagy interferes with T cell receptor signalling remains to be fully determined. Primary investigations of the signalling events upon T cell receptor engagement in autophagy-deficient cells have revealed impaired mTOR and AMPK activity [92]. This goes together with the observation that the increase in the cellular ATP levels, which is expected upon T cell activation, could not be detected [92]. The finding that a low rate of ATP generation occurs together with decreased fatty acid usage and impaired AMPK activation strongly implicates AMPK-induced autophagy as an essential process for breaking down intracellular lipids for ATP production during T cell activation [92]. These findings suggest that the activation of naïve T cells may require initial AMPK-induced autophagy to sustain the first wave of energy demands prior to the upregulation of metabolite transporters and metabolic enzymes needed for growth and proliferation.

Another functional role of autophagy has been demonstrated in effector CD4+ T helper 1 cells. These cells were found to have a profound defect in their ability to secrete IL-2 when autophagy was blocked during stimulation [92]. Notably, the deficiency in autophagy did not interfere with the process of IL-2 secretion but rather with the synthesis of IL-2 itself. Interestingly, IL-2 expression was restored with methyl-pyruvate addition, suggesting that the decreased capability of autophagy-deficient T cells to fuel metabolism does not only impair their proliferation but also impairs their effector functions.

In recent years, the autophagy suppressor mTOR has been in the research spotlight because of its cell-intrinsic role for memory T cell differentiation. Inhibition of mTOR by rapamycin during T cell activation and proliferation results in an enhanced frequency of CD8+ memory T cells [93]. This effect was determined to be mediated by the ability of mTOR to influence the transcription factors t-bet and eomesodermin, which were shown to be crucial in determining effector T cell fate [94]. However, the augmented CD8+ memory T cell response upon mTOR inhibition was also shown to be associated with changes in metabolism [95]. Rapamycin-treated effector T cells gained ATP mainly through oxidative phosphorylation and exhibited a memory precursor like phenotype which could allow for long-lasting antitumor immunity. These cells were also more resistant to growth factor withdrawal-induced cell death. The finding that memory precursor cells were generated by switching their metabolism to oxidative phosphorylation underscores the hypothesis that the metabolic characteristics of effector cells are crucial for the formation of CD8 memory [95]. Although these results were not discussed in connection with the effect of mTOR inhibition on the induction of autophagy, a role of autophagy in the fate decision of memory T cells is a possibility.

## 4. Conclusion

**Figure 4:**
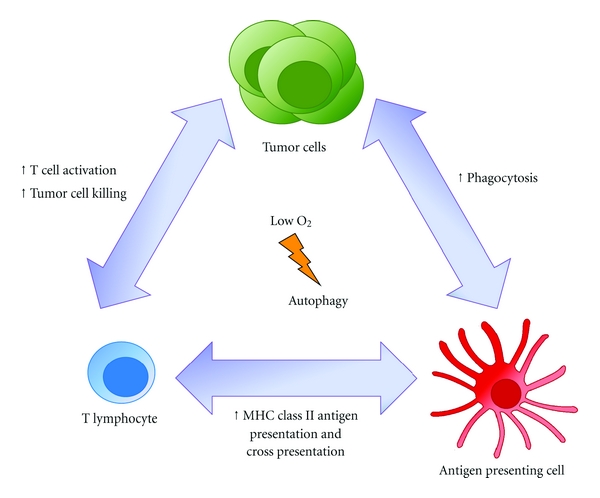
Proposed roles of autophagy under hypoxia in tumor immunology. Autophagy induction may improve phagocytosis maturation by enhancing degradation of tumor cells by innate immune cells. Autophagy aids in both the cross presentation of exogenous antigens on MHC class I and presentation of endogenous antigens on MHC class II. In T lymphocytes, autophagy enables activation by providing available nutrients from the degradation of macromolecules. It also enables cytotoxic cytokine secretion which enhances tumor cell killing. All cell types, including both immune cells and tumor cells, may utilize autophagy to enhance survival under hypoxic and nutrient deprived conditions.

The tumor microenvironment is complex, comprised of a variety of factors that can act on all cells including immune and cancer cells (Figure 4). One of the predominant factors is hypoxia, which is a known inducer of autophagy. Though hypoxia-induced autophagy promotes cellular survival and function, hypoxia-induced autophagy has also been demonstrated to negatively impact the viability of specific tumor types. In order to reconcile this disparate observation, further investigation is required to understand what factors dictate whether autophagy enhances or suppresses cellular viability during hypoxia. This is paramount since numerous drugs that target the hypoxia pathway have been deployed to treat cancer and their impact on autophagy has not been examined. For example, agents such as Avastin*™* reduce the formation of new blood vessels in hypoxic tumors [96], but may indirectly promote tumor cell autophagy and survival. Moreover, despite the widespread belief that mTOR controls tumor growth and proliferation, clinical trials with mTOR inhibitors have been disappointing [97, 98]. It could be argued that the ineffectiveness of mTOR blockage is due to autophagy activation and therefore this approach should be combined with a suitable inhibitor of autophagy. This idea is supported by preclinical data demonstrating the synergistic effect of autophagy-inducing agents (chemotherapy, radiation) and the use of an inhibitor of autophagy in cells reliant on this survival pathway. Several studies in human prostate, colon, breast, and lung cancers are now underway to examine combined chemotherapy and autophagy inhibition using the antimalarial hydroxychloroquine (NCT00786682, NCT01206530, NCT01006369, NCT00765765, NCT00933803 reviewed by [99, 100]).

The key question is whether autophagy inhibition in the clinical setting will prove to be specific enough to selectively kill tumor cells. Since there is mounting evidence to suggest that autophagy induction enhances immune cell function, therapeutic strategies targeting this pathway must take into account the potential negative impact on antitumor immunity. Given that the immune system is a powerful foe to cancer, therapies that do little to dampen the antitumor immune response, or preferably enhance the response would be optimal. Further research will be essential for determining how best to modulate autophagy in cancer patients with this goal in mind.

## Abbreviations

AICD: Activation-induced cell death
AMP: Adenosine monophosphate
AMPK: Adenosine monophosphate-activated protein kinase
ATF4: Activating transcription factor 4
ATG: Autophagy-related gene
ATP: Adenosine triphosphate
APC: Antigen presenting cell
Bcl-xL: B-cell lymphoma-extra large
Bcl-2: B-cell lymphoma 2
BNIP3: Bcl-2/adenovirus E1B 19-kDa interacting protein 3
BNIP3L: BNIP3-like
DC: Dendritic cell
ER: Endoplasmic reticulum
FoxP3: Forkhead box P3
HIF-1: Hypoxia inducible factor 1
HMGB1: High mobility group box-1
IL: Interleukin
LC3: Microtubule-associated light chain 3
MEF: Mouse embryonic fibroblast
MHC: Major histocompatibility complex
mTOR: Mammalian target of rapamycin
MyD88: Myeloid differentiation primary response gene 88
OxPhos: Oxidative phosphorylation
PERK: PKR-like ER kinase
PMA: Phorbol myristate acetate
ROS: Reactive oxygen species
TCR: T cell receptor
TLR: Toll-Like receptor
TRIF: Toll/interleukin-1 receptor-domain-containing adapter-inducing interferon-*β*
UPR: Unfolded protein response.
